# Explanation

**Published:** 1844-12

**Authors:** 


### Miscellaneous Notices.

*Explanation.*—It having been stated by some of the readers of the Journal, and with some degree of prejudice and blame to the Washington editor, that the remarks made by him in his "gossip," of the last March number, as to the manner in which some men get business, were uncalled for, as far as they alluded to two persons, who have within the last two years associated themselves together, and in justice to all parties, we make the following explanation:

Dr. J. Smith Dodge, late of the Bowery, but now of Bond street, New York, in 1842, employed as an assistant in mechanical dentistry, a person who was *then* known as Luther *Parmelee,* late machinist, and in some few months afterwards there appeared on a plate in Bond street, "Dodge & *Parmely,* dentists." During the ensuing session of congress, there also appeared in the newspapers an advertisement of "Dodge & Parmely, dentists, of Bond street, New York," offering their services to the citizens, &c. of Washington. In conversation, Dr Dodge stated to the Washington editor, that his partner was not either Dr. E. or Dr. J. Parmly, of Bond street, "but one of the same family;" and it was upon hearing from the Drs. Parmly, subsequently, that they had never seen Dr. Dodge's partner nor known any thing of him, farther than hearing from Dr. D., that such a person was with him, that the remarks alluded to were penned. Since then some correspondence has taken place between the parties, which, as we recently learned while in New York, resulted in another change, and the names now stand "Dodge & *Parmele.*" We would, in justice to Dr. Dodge, state, that we are credibly informed that he did not *make* the first change in his partner's name; but it is in evidence that he knew the change had been made when he used it in connection with his own.

We have not yet heard what reason Dr. D. assigns for giving in his circular as his partner *doctor* L. Parmely, or for stating to the Washington editor that his partner was "one of the same family" as the Parmlys, as we believe that the parties themselves neither know of or claim any relationship with each other.

With this explanation, we are willing to bear any blame that may attach to the appearance of the remarks in the Journal, that have been complained of by those unacquainted with the circumstances.

The above was in type for the September number of the Journal, but was omitted for want of room.—*Eds.*

